# What are the determinants of antiretroviral therapy adherence among stable people living with HIV? A cross-sectional study in Cambodia

**DOI:** 10.1186/s12981-023-00544-w

**Published:** 2023-07-14

**Authors:** Sovannary Tuot, Jian Wei Sim, Michiko Nagashima-Hayashi, Pheak Chhoun, Alvin Kuo Jing Teo, Kiesha Prem, Siyan Yi

**Affiliations:** 1 KHANA Center for Population Health Research, Phnom Penh, Cambodia; 2 Department of Community and Global Health, Graduate School of Medicine, The University of Tokyo, Tokyo, Japan; 3 Faculty of Social Sciences and Humanity, Royal University of Phnom Penh, Phnom Penh, Cambodia; 4 Saw Swee Hock School of Public Health, National University of Singapore and National University Health System, 12 Science Drive 2, #10- 01, 117549 Singapore, Singapore; 5 Faculty of Medicine and Health, University of Sydney, Sydney, NSW Australia; 6 Department of Infectious Disease Epidemiology, London School of Hygiene & Tropical Medicine, London, UK; 7 Center for Global Health Research, Public Health Program, Touro University California, Vallejo, CA USA

**Keywords:** AIDS, People living with HIV, Antiretroviral therapy, Adherence, Treatment outcome, Asia

## Abstract

**Background:**

Understanding context-specific determinants of antiretroviral therapy (ART) adherence is crucial for developing tailored interventions for improving health outcomes and achieving the UNAIDS’ third 95% target. This cross-sectional study explores factors associated with ART adherence among stable people living with HIV on ART in Cambodia.

**Methods:**

We used baseline survey data from a quasi-experimental study conducted in 2021. The participants were recruited from 20 ART clinics in nine provinces for face-to-face interviews. A structured questionnaire collected information on sociodemographic characteristics, ART adherence, perceived ART self-efficacy, mental health, quality of life, stigma, and discrimination. We conducted bivariate and multiple logistic regression analyses to identify factors associated with ART adherence.

**Results:**

Out of the 4101 participants, 86.5% reported adhering to ART in the past two months. The adjusted odds of ART adherence were significantly higher among participants in older age groups than those aged 15–29, participants with elevated cholesterol than those without it, participants who exhibited strong self-efficacy in health responsibility to maintain life than those with poor self-efficacy in health responsibility, participants who scored < 3 on the stigma and discrimination scale than those who scored ≥ 3, participants who scored ≥ 42 on the mental component of the quality-of-life scale than those who scored < 42. The adjusted odds of ART adherence were significantly lower in participants who earned > 301 USD per month than those who earned ≤ 100 USD per month.

**Conclusion:**

The ART adherence rate among stable people living with HIV in this study was comparable to that of the general people living with HIV in Cambodia. The results suggest the need for innovative interventions to further reduce stigma and discrimination and strategies to improve the self-efficacy and mental health of people living with HIV to improve ART adherence.

## Introduction

Significant progress has been made in the battle against the HIV epidemic in the past two decades. Adherence to antiretroviral therapy (ART) has become the key for AIDS to become a manageable chronic disease [1]. Based on these developments, the Joint United Nations Program on HIV/AIDS (UNAIDS) launched a Fast-Track strategy in 2014. The strategy intends to end the global AIDS epidemic by achieving the 95-95-95 targets by 2030 [2] – i.e., to diagnose 95% of all people living with HIV, provide ART for 95% of those diagnosed with HIV, and achieve viral suppression for 95% of people who receive the ART.

Non-adherence to ART is the main reason for treatment failure, resulting in individuals with HIV progressing to AIDS despite being enrolled in the ART program [3]. ART adherence, defined by the patient’s ability to follow the medication regimen and diet restrictions, must be at 70–90% to effectively suppress viral load, thus reducing the risk of transmitting HIV to another person [4]. Poor adherence can also lead to higher hospitalization rates and productivity losses [5]. It may also lead to drug resistance against first-line antiretrovirals (ARVs) [6], raising the demand for second or third-line ARVs that are often more costly and with higher pill burden, which is often associated with poorer health outcomes [7].

Studies from various countries have identified factors associated with ART adherence. For example, being male, being younger, fear of stigma, alcohol and substance use, and lower income have been associated with suboptimal ART adherence [8–12]. These factors can be grouped into four main categories – (i) intrapersonal, (ii) interpersonal, (iii) medication-related, and (iv) healthcare systems-related factors [13]. Intrapersonal factors refer to an individual’s demographics, psychosocial elements, personal beliefs, and health status. Interpersonal-related factors include relationships between patients and their families, caregivers, and healthcare providers. While family and community support positively influence ART initiation and adherence in people living with HIV, lack thereof and stigma in the family and community can result in poor adherence [14, 15]. Similarly, the attitude of healthcare providers can influence the motivation of people living with HIV for ART adherence both positively and negatively [16–18]. Medication-related factors, such as patients’ perception of the effectiveness of ART and pill burden, affect treatment adherence and continuation [3]. Health systems-related factors refer to the availability and accessibility to ART, including out-of-pocket ARV costs, distance and transportation costs to reach the services, and health service quality, such as long waiting time [13, 14].

In Cambodia, the estimated number of people living with HIV in 2020 was around 75,000, with an HIV prevalence of 0.6% among the general population aged 15–49 [19]. As of 2020, Cambodia was one of the three countries that have successfully achieved the 90-90-90 HIV targets, with 80% of all people living with HIV having their viral load suppressed. Given this trend, the country now strives to achieve the third UNAIDS target, i.e., further increasing the number of people living with HIV with suppressed viral load. There is an urgent need to understand factors associated with ART non-adherence, even among stable people living with HIV, to develop context-specific interventions to address them. Although the target population was limited to adolescents, a cross-sectional study conducted in 2018 among those aged 15–17 in Cambodia found that the probability of having viral suppression was significantly higher among participants who were older, had been on ART for a more extended period, had a higher CD4 count at the beginning of ART, had a parent as their main daily caregiver, and did not believe that there was a cure for AIDS [20].

Noting that the associated factors of ART adherence can vary among different populations [21], in this present study, we aimed to explore the ART adherence level and identify its associated factors among stable adults living with HIV. To date, no studies have examined ART adherence levels and related factors among this population in Cambodia. Given the scant evidence in low- and middle-income countries, identifying the determinants that hinder their adherence would help provide valuable insights into the underlying factors of ART adherence among stable people living with HIV in Cambodia and other resource-constraint settings.

## Methods

### Study design and setting

This cross-sectional study used the baseline data from a quasi-experimental study conducted in July 2021 before the intervention began in November of the same year. Implemented by Khmer HIV/AIDS NGO Alliance (KHANA), a leading non-governmental organization working on HIV and AIDS in Cambodia, the parent study aims to assess the feasibility and efficiency of a community-based ART delivery (CAD) model in bringing ART closer to the people living with HIV and improving social support in the community. The medications are distributed during the monthly community meetings, where the members of the community ART group would interact with their peers for medical, social, and emotional support. The model is expected to sustain the treatment outcomes and reduce the economic burden, stigma, and discrimination among stable people living with HIV and the workload of healthcare workers at ART clinics. The intervention study’s protocol has been published elsewhere [22].

We developed a list of participating ART clinics after consulting the Database Management Unit of the National Center for HIV/AIDS, Dermatology, and STD (NCHADS) and other partners involved in the HIV program implementation. We selected 20 ART clinics in 10 provinces (10 clinics for intervention and 10 for the control arm) for the parent study based on the availability of the implementing partners and the number of stable people living with HIV eligible for enrolment.

### Participant recruitment

This study included a total sample of 4101 participants . The study team and on-site clinicians assessed and enrolled the participants using the eligibility criteria of stable people living with HIV during their visits to ART clinics. The criteria used to define stable people living with HIV on ART were: (1) ≥ 15 years old, (2) have received first-line ART for at least one year, (3) had not reported any adverse reactions related to ART or drug interactions that require regular monitoring in the past three months, (4) were not having tuberculosis or other opportunistic infections and not taking any prophylactic treatment; (5) were not pregnant or breastfeeding (for women); (6) had a good understanding of lifelong treatment and adherence to medication, evaluated by the ART clinic physicians using two questions on how to remember and plan their daily ARVs; and (7) had achieved treatment success – two consecutive undetectable viral loads or CD4 counts above 200 cells/mm^3^ [23]. Field workers and coordinators then approached all eligible people to obtain consent to participate in the study and included all consented people. The data collection team double-checked each questionnaire after the interview. The questionnaire would be returned to the interviewer if any information needed to be included or confirmed before the participant left the site.

### Variables and measurements

In this analysis, we included the following variables extracted from the baseline survey of the CAD study: ART adherence, sociodemographic characteristics, depressive symptoms, quality of life (QoL), AIDS-related stigma and discrimination, perceived self-efficacy, and social support.

There is no standard method for measuring ART adherence [24]. The World Health Organization (WHO) recommends that a multi-method approach be used to measure adherence levels [25]. In this study, we employed self-reporting adherence questions, the Visual Analogue Scale (VAS), and the knowledge of pill identification test to assess adherence [26]. The self-reporting method has been consistently associated with clinical outcomes and is widely used for its low cost and ease of implementation [27, 28]. Self-reporting adherence questions require participants to answer if they had missed any ART dose in the past week, the reason for missing them, and their perception of their health status after taking them [26]. The questionnaire included seven questions, and the participants would be considered “good adherent” if they answered “no” to all questions and “moderately or poorly adherent” if they answered “yes” to any of the questions. VAS questions asked participants to recall if they had missed any dose over the past four days with a percentage adherence scale. Participants with a result above 95% would be considered adherent, while those who scored 95% and below would be regarded as non-adherent [29].

For the knowledge of pill identification test, participants were asked to show their medication and if they could remember the dosage, the frequency, prescribed timing, and other additional instructions. Only participants who followed the prescription as intended would be considered adherent. We calculated each adherence method separately before combining them to determine the adherence level. Participants would be regarded as ‘adherent to ART’ if they reported that they adhered to ART in the past two months, had a VAS score > 95%, and could correctly answer all questions in the knowledge of the pill identification test.

Sociodemographic characteristics included age group, sex, marital status, employment, monthly income, and formal education level. HIV treatment history and health status information included ART treatment duration, transportation mode, relationship with healthcare providers and satisfaction with healthcare services, and comorbidities of other chronic diseases such as diabetes mellitus, elevated cholesterol, and hypertension. We measured healthcare service satisfaction by asking whether the participants were satisfied with the healthcare services they received at the ART clinic with Likert scale response options (very dissatisfied, dissatisfied, neither satisfied nor dissatisfied, satisfied, and very satisfied). We also asked participants to rate their overall relationship with healthcare providers at the ART clinic on a Likert scale response options (good, fair, poor, and other or no response).

We measured depressive symptoms using the Center for Epidemiologic Studies Depression Scale (CES-D-10), a 10-item four-point Likert scale questionnaire assessing depressive symptoms (Cronbach’s alpha = 0.78). A cut-off score of 10 or higher suggests significant depressive symptoms [30, 31]. We used the 12-item Short Form Survey (SF-12) to assess health-related QoL, generating two 0-100 component scores – physical (PCS, Cronbach’s alpha = 0.69) and mental (MCS, Cronbach’s alpha = 0.59). A score of 50 or less on the PCS indicates a physical condition, and a score of 42 or less on the MCS indicates clinical depression [32–34].

We used the People Living with HIV Stigma Index to measure AIDS-related stigma and discrimination experienced over the past 12 months [35–37]. The questionnaire has three main sections: [1] experiences of AIDS-related stigma and discrimination in various settings such as home, community, workplace, and religious settings; [2] internal AIDS-related stigma; and [3] fear of AIDS-related stigma and discrimination from family and communities (Cronbach’s alpha = 0.75). The total score was calculated in each section, and the mean score of each section was used to divide the participants into two groups, one with lower and another with higher AIDS-related stigma and discrimination [35].

We measured self-efficacy using the Perceived Self-Efficacy for Receiving Antiretroviral Therapy Scale (PSEARTS), which assesses the participant’s confidence in carrying out health-related behaviors [38, 39]. PSEARTS has five components: [1] self-efficacy in health responsibility to maintain life (Cronbach’s alpha = 0.70), [2] self-efficacy in physical activities for life (Cronbach’s alpha = 0.82), [3] self-efficacy in nutrition for life (Cronbach’s alpha = 0.71), [4] self-efficacy in spiritual growth for life (Cronbach’s alpha = 0.73), and [5] self-efficacy in stress management for life (Cronbach’s alpha = 0.79). The questionnaire is a four-point Likert scale based on confidence levels (no, low, high, and very high confidence). The participants were then categorized into poor (no/low confidence) and strong self-efficacy (high and very high confidence).

Social support was assessed by the Berlin Social Support Scale (BSSS), a 4-point Likert scale questionnaire. The total possible scores were 12–60, with a higher score indicating better perceived social support (Cronbach’s alpha = 0.89). The scores were further categorized into low (12–27 scores), moderate (28–44 scores), and high (45–60 scores) perceived social support [40–42].

### Data analyses

We stored collected data in REDCap and used Stata 16 (Stata Corp LP, College Station, TX, USA) for analyses. We conducted bivariate analyses using Pearson’s Chi-square test to compare categorical variables and Student’s *t*-test to examine continuous variables. Bivariable logistic regression analyses were used to identify factors associated with ART adherence. We constructed a multiple logistic model, including variables associated with ART adherence at *p*-value < 0.1 in bivariate logistic regression analyses in the model. A backward stepwise method was implemented to remove variables with *p* > 0.05 one by one from the model. Adjusted odds ratios (AOR) with 95% confidence intervals (CI) and two-tailed *p*-values were reported.

### Ethical consideration

Participants were informed about the study’s objectives and the participation risks and benefits before enrolling them in the study. If a participant could not read or write, data collectors would read the information sheet during the consent-taking process. Participants could withdraw from or discontinue the study at any time. Participants’ written, recorded, and transcribed data were stored securely. We assigned codes to participants’ identifiers, and data collection was conducted in a private room. After completing the interview, participants received US$5 for their time and transportation compensation.

## Results

### Sociodemographic characteristics

Table 1 shows that 41.4% of the participants were male, and 78.7% were ≥ 40 years old. More than half (59.0%) of the participants were married, and 66.0% had only a primary school education or lower. About one in four (26.2%) were farmers or fishermen, and 18.8% were self-employed. Almost half (48.1%) earned less than 100 USD per month, and 50.0% traveled less than an hour to the ART clinic.

**Table 1:** Sociodemographic characteristics, comorbidities, health service satisfaction, mental health, and quality of life of stable people living with HIV (n = 4101)

Sociodemographic characteristics	Frequency (%)
Female	2402 (58.6)
Age group	
15–29	380 (9.3)
30–39	493 (12.0)
40–49	1584 (38.6)
50–59	1240 (30.2)
60–82	404 (9.9)
Marital status	
Never married	429 (10.5)
Married	2421 (59.0)
Divorced/widowed/other	1251 (30.5)
Formal education level	
No formal education (0 years)	727 (17.7)
Primary school (1 to 6 years)	1980 (48.3)
Junior high (7 to 9 years)	848 (20.7)
High school (10 to 12 years)	386 (9.4)
University (≥13 years)	160 (3.9)
Employment	
Unemployed	769 (18.8)
Farmer/fisherman	1074 (26.2)
Self-employed business	770 (18.8)
Government staff/uniformed officer (policeman, soldier)	171 (4.1)
Private employee/non-governmental organization staff	331 (8.1)
Construction worker	617 (15.1)
Motor/taxi driver/other	369 (9.0)
Monthly income (USD)	
0 to 100	1974 (48.1)
101 to 200	1289 (31.4)
201 to 300	466 (11.4)
301 and above	372 (9.1)
ART duration	
1 to 5 years	723 (17.6)
6 to 10 years	852 (20.8)
≥ 11 years	2526 (61.6)
Mode of transport to access ART clinic	
Bicycle	121 (3.0)
Tuk-tuk	265 (6.4)
Motorcycle	3102 (75.6)
Car	490 (11.9)
On foot/ boat/ship/other	123 (3.1)
Patient satisfaction with healthcare services	
Very dissatisfied/dissatisfied	61 (1.5)
Neither satisfied nor dissatisfied	182 (4.4)
Very satisfied/satisfied	3858 (94.1)
Relationship with healthcare providers	
Good	3630 (88.5)
Fair	457 (11.1)
Bad	14 (0.3)
Having diabetes mellitus	320 (7.8)
Having hypertension	692 (16.9)
Having elevated cholesterol	272 (6.6)
Traveling time (≥ 1 h)	2050 (50.0)
Clinic waiting time (≥ 2 h)	2014 (49.1)
Poor self-efficacy in health responsibility to maintain life	1178 (28.7)
Poor self-efficacy in physical activities for life	1739 (42.4)
Poor self-efficacy in nutrition for life	1181 (28.8)
Poor self-efficacy in spiritual growth for life	1129 (27.5)
Poor self-efficacy in stress management for life	921 (22.5)
Poor overall self-efficacy	2671 (65.1)
Depressive symptoms	800 (19.5)
Low perceived social support	22 (0.5)
Moderate perceived social support	288 (7.0)
High perceived social support	3791 (92.4)
Total score of AIDS-related stigma and discrimination experienced ≤ 2^*^	2724 (66.4)
Total score of internal AIDS-related stigma ≤ 4^*^	3822 (93.2)
Total score of fear of AIDS-related stigma and discrimination ≤ 1^*^	3568 (87.0)
Quality of life (physical component summary) < 50^†^	1537 (37.5)
Quality of life (mental component summary) < 42^†^	830 (20.2)

*HIV, human immunodeficiency virus; USD, the United States dollar.*

^*^ *Total scores for the three main categories of stigma and discrimination were tabulated using the People Living with HIV Stigma Scale. A total score of stigma and discrimination ≤ 2 indicates low stigma and discrimination. A total score of internal stigma ≤ 4 indicates low internal stigma. A total score of fear of stigma and discrimination ≤ 1 indicates low fear of stigma and discrimination.*

^*†*^ *A score of ≤ 50 on the physical condition scale indicates a physical condition, and ≤ 42 on the mental condition scale indicates clinical depression.*

Table 1 also shows that 61.6% of the study participants had been on ART for ≥ 11 years. The majority (94.1%) were either satisfied or very satisfied with the healthcare services, with 88.5% reporting having good relationships with their healthcare providers. Regarding comorbidities, 7.8% reported having diabetes mellitus, 16.9% having hypertension, and 6.6% having elevated cholesterol. About two-thirds (65.1%) had poor self-efficacy, and 19.5% had a possible risk of clinical depression. The majority (92.4%) expressed high perceived social support. About one-third (33.6%) had experienced high AIDS-related stigma and discrimination, 6.8% had a high internal AIDS-related stigma, and 13% feared AIDS-related stigma and discrimination over the past 12 months. Over one-third (37.5%) had poor physical QoL, and 20.2% had poor mental QoL.

### Antiretroviral therapy (ART) adherence

As shown in Tables 2, 88.2% of the participants reported adhering to ART, 97.1% had a VAS score > 95%, and 98.1% reported being able to take the medication as prescribed. The multi-method approach combining all three methods showed that 86.5% of the participants adhered to ART.

**Table 2:** Adherence to ART among stable people living with HIV (n = 4101)

Assessment of ART adherence	Frequency (%)
Self-reporting adherence	
Non-adherent	484 (11.8)
Adherent	3617 (88.2)
Visual analogue scale	
Visual analogue scale score ≤ 95	120 (2.9)
Visual analogue scale score > 95	3981 (97.1)
Knowledge of pill identification test	
No	77 (1.9)
Yes	4024 (98.1)
Combined method^*^	
Non-adherent	554 (13.5)
Adherent	3547 (86.5)

*ART, antiretroviral therapy; HIV, human immunodeficiency virus.*

^*^ *Participants would be considered ‘adherent to ART’ if they reported that they adhered to ART, had the visual analogue scale score > 95, and could correctly answer all questions in the knowledge of the pill identification test.*

### Bivariate logistic regression

Table 3 shows that older participants were significantly more likely to adhere to ART than those younger than 30. Married participants were significantly more likely to adhere to ART than those who never married. Participants with a monthly income of ≤ 100 USD were significantly more likely to adhere to ART than those who earned ≥ 301 USD. Participants on ART for > 5 years were significantly more likely to adhere to ART than those on ART for ≤ 5 years. Participants with diabetes or elevated cholesterol were significantly more likely to adhere to ART than those who did not have these chronic comorbidities.

**Table 3:** Bivariable logistic regression analysis of factors associated with ART adherence among stable people living with HIV (n = 4101)

Variables		Adherent (*n* = 3547)	Non-adherent (*n* = 554)	OR (95% CI)
Female		2071 (50.5)	331 (8.1)	0.95 (0.79, 1.13)
Age group (years)				
	15–29	76 (1.9)	304 (7.4)	1
	30–39	89 (2.2)	404 (9.9)	1.13 (0.81, 1.60)
	40–49	198 (4.8)	1386 (33.8)	1.75 (1.31, 2.34) ^***^
	50–59	150 (3.7)	1090 (26.6)	1.82 (1.34, 2.46)^***^
	60–82	41 (1.0)	363 (8.9)	2.21 (3.11, 5.14)^***^
Marital status				
	Never married	81 (2.0)	348 (8.5)	1
	Married	298 (7.3)	2123 (51.8)	1.66 (1.27, 2.17)^***^
	Divorced/widowed/other	175 (4.3)	1076 (26.2)	1.43 (1.07, 1.91)^*^
Education level				
	No formal education (0 years)	96 (2.3)	631 (15.4)	1
	Primary school (1–6 years)	260 (6.3)	1720 (41.9)	1.00 (0.78, 1.29)
	Junior high (7–9 years)	113 (2.8)	735 (17.9)	0.99 (0.74, 1.33)
	High school (10–12 years)	55 (1.3)	331 (8.1)	0.92 (0.64, 1.31)
	Degree and above (≥ 13 years)	30 (0.7)	130 (3.2)	0.66 (0.42, 1.04)
Occupation				
	Unemployed	101 (2.5)	668 (16.3)	1
	Farmer/fisherman	133 (3.2)	941 (22.9)	1.07 (0.81, 1.41)
	Self-employed business	109 (2.7)	661 (16.1)	0.92 (0.69, 1.23)
	Government staff/uniformed officer	24 (0.6)	147 (3.6)	0.93 (0.57, 1.50)
	Private employee/NGO staff	53 (1.3)	278 (6.8)	0.79 (0.55, 1.14)
	Construction worker	86 (2.1)	531 (12.9)	0.93 (0.69, 1.27)
	Motor/taxi driver/others	48 (1.2)	321 (7.8)	1.01 (0.70, 1.46)
Monthly income (USD)				
	0–100	247 (6.0)	1727 (42.1)	1
	101–200	173 (4.2)	1116 (27.2)	0.92 (0.75, 1.14)
	201–300	56 (1.4)	410 (10.0)	1.05 (0.77, 1.43)
	> 300	78 (1.9)	294 (7.2)	0.54 (0.41, 0.72)^***^
ART duration (years)				
	1–5	120 (2.9)	603 (14.7)	1
	6–10	106 (2.6)	746 (18.2)	1.40 (1.06, 1.86)^*^
	> 10	328 (8.0)	2198 (53.6)	1.33 (1.06, 1.67)^*^
Mode of transport to access ART clinic				
	Bicycle	19 (0.5)	102 (2.5)	0.75 (0.36, 1.55)
	Tuk-tuk	51 (1.2)	214 (5.2)	0.58 (0.31, 1.08)
	Motorcycle	399 (9.7)	2703 (65.9)	0.94 (0.54, 1.63)
	Car	70 (1.7)	420 (10.2)	0.83 (0.46, 1.51)
	On foot/ boat/ship/other	15 (0.4)	108 (2.6)	1
Patient satisfaction with healthcare services				
	Very dissatisfied/dissatisfied	15 (0.4)	46 (1.1)	1
	Neither satisfied nor dissatisfied	29 (0.7)	153 (3.7)	0.65 (0.51, 0.84)
	Very satisfied/satisfied	510 (12.4)	3348 (81.6)	0.37 (0.12, 1.18)
Relationship with healthcare providers				
	Good	466 (11.4)	3164 (77.2)	1
	Fair	84 (2.0)	373 (9.1)	0.65 (0.51, 0.84)^**^
	Bad	4 (0.1)	10 (0.2)	0.37 (0.12, 1.18)
Having diabetes mellitus		3288 (80.2)	493 (12.0)	1.57 (1.17, 2.11)^**^
Having hypertension		2954 (72.0)	455 (11.1)	1.08 (0.86, 1.37)
Having elevated cholesterol		3330 (81.2)	499 (12.2)	1.69 (1.24, 2.31)^**^
Travel time to ART clinic (≥ 1 h)		1762 (43.0)	288 (7.0)	0.91 (0.76, 1.09)
Waiting time at the ART clinic (≥ 2 h)		1744 (45.0)	270 (6.6)	1.02 (5.60, 7.19)
Strong self-efficacy in health responsibility to maintain life		2603 (63.5)	320 (7.8)	2.02 (1.68, 2.42)^***^
Strong self-efficacy in physical activities		2062 (50.3)	300 (7.3)	1.18 (0.98, 1.41)
Strong self-efficacy in nutrition		2552 (62.2)	368 (9.0)	1.30 (1.07, 1.57)^***^
Strong self-efficacy in spiritual growth		2598 (63.4)	374 (9.1)	1.32 (1.09, 1.60)^***^
Strong self-efficacy in stress management		2796 (68.2)	384 (9.4)	1.65 (1.35, 2.01)^***^
Strong overall self-efficacy		1269 (30.9)	161 (3.9)	1.36 (1.12, 1.65)^***^
No depressive symptoms		2914 (71.1)	387 (9.4)	1.99 (1.63, 2.43)^***^
Perceived social support				
	Low	7 (0.2)	15 (0.4)	1
	Moderate	52 (1.3)	236 (5.8)	2.12 (0.82, 5.45)
	High	495 (12.1)	3296 (80.4)	3.11 (1.26, 7.66)^*^
Total AIDS-related stigma and discrimination score ≤ 2		3345 (81.6)	477 (11.6)	2.67 (2.02, 3.54)^***^
Total score of internal AIDS-related stigma ≤ 4		2406 (58.7)	318 (7.8)	1.56 (1.30, 1.88)^***^
Total fear of AIDS-related stigma and discrimination score ≤ 1		3128 (76.3)	440 (10.7)	1.93 (1.54, 2.43)^***^
Total quality of life score (physical component summary) ≥ 50		2264 (55.2)	300 (7.3)	1.49 (1.25, 1.79)^***^
Total quality of life score (mental component summary) ≥ 42		2894 (70.6)	377 (9.2)	2.08 (1.71, 2.54)^***^

*ART, antiretroviral therapy; CI, confidence interval, HIV, human immunodeficiency virus, NGO, non-governmental organization; OR, odd ratio; USD, United States dollar.*

^*^P < 0.05, ^**^P < 0.01, ^***^P < 0.001.

As shown in Table 3, participants with good relationships with their healthcare providers were significantly more likely to adhere to ART than those with fair or poor relationships. Participants with strong self-efficacies in health responsibility to maintain life, nutrition, spiritual growth, and stress management were more likely to adhere to ART than those with poor self-efficacy.

Participants without depressive symptoms were significantly more likely to adhere to ART than those with depressive symptoms. Participants with high perceived social support were significantly more likely to adhere to ART than those with low perceived social support. Participants who reported lower AIDS-related stigma and discrimination scores, lower internal AIDS-related stigma scores, and lower fears of AIDS-related stigma and discrimination were significantly more likely to adhere to ART than those who reported higher AIDS-related stigma and discrimination scores, higher internal AIDS-related stigma scores, and higher fears of AIDS-related stigma and discrimination, respectively. Participants with higher PCS and MCS scores were significantly more likely to adhere to ART than those who had lower PCS and MCS scores, respectively.

### Multiple logistic regression model

Table 4 shows that the adjusted odds of ART adherence were significantly higher among participants in older age groups than those aged 15–29, participants with elevated cholesterol than those without it, participants who exhibited strong self-efficacy in health responsibility to maintain life than those with poor self-efficacy in health responsibility to maintain life, participants who scored < 3 on the AIDS-related stigma and discrimination scale than those who scored ≥ 3, participants who scored ≥ 42 on the mental component of the QoL scale than those who scored < 42. The adjusted odds of ART adherence were significantly lower in participants who earned > 301 USD per month than those who earned ≤ 100 USD per month.

**Table 4:** Final model of the multiple logistic regression analysis of factors associated with ART adherence among stable people living with HIV (*n* = 4101)

Variables in the final model		AOR (95% CI)	*P*-value
Age group (years)			
	15–29	1	
	30–39	1.23 (0.87, 1.75)	0.24
	40–49	1.74 (1.28, 2.35)	< 0.001
	50–59	1.87 (1.36, 2.56)	< 0.001
	60–82	2.11 (1.39, 3.22)	0.001
Monthly income (USD)			
	0–100	1	
	101–200	0.83 (0.67, 1.04)	0.10
	201–300	0.89 (0.65, 1.23)	0.49
	> 300	0.49 (0.37, 0.66)	< 0.001
Having elevated cholesterol			
	No	1	
	Yes	1.61 (1.16, 2.23)	0.004
Self-efficacy in health responsibility to maintain life			
	Poor	1	
	Strong	1.83 (1.51, 2.22)	< 0.001
Total AIDS-related stigma and discrimination score			
	< 3	1.87 (1.38, 2.54)	< 0.001
	≥ 3	1	
Total internal AIDS-related stigma score			
	< 5	1.39 (1.14, 1.70)	0.001
	≥ 5	1	
Total quality of life score (mental component summary) ≥ 42			
	< 42	1	
	≥ 42	1.60 (1.28, 1.99)	< 0.001

*AOR, adjusted odd ratio; ART, antiretroviral therapy; CI, confidence interval, HIV, human immunodeficiency virus, USD, United States dollar.*

*Variables associated with ART adherence in bivariable logistic regression analysis at p ≤ 0.1 were included in the multiple logistic regression model.*

## Discussion

This study examined ART adherence and its associated factors among stable people living with HIV as part of a CAD implementation program in Cambodia. The ART adherence rate among stable people living with HIV in this study is comparable to that of the general people living with HIV in Cambodia, where 80% of all people living with HIV have viral suppression [19]. Age, monthly income, cholesterol level, self-efficacy in health responsibility to maintain life, AIDS-related stigma and discrimination, and mental health were significantly associated with ART adherence.

Participants aged 40 and older were more likely to adhere to ART than those younger than 30. This finding is similar to our previous study, where older adolescents were more likely to have viral suppression than the younger groups [20]. This result supports other studies where better adherence rates have been observed in older people living with HIV [8, 9]. It indicates the need for tailored interventions to promote adherence among adolescents and young adults living with HIV. Regarding income, participants with a monthly income of US$300 or more were less likely to adhere to ART than those earning $100 and less. One of the possible explanations could be that, in Cambodia, ART is provided free of charge for everyone [40], and the government and NGOs target poorer and more vulnerable groups for non-clinical interventions. Hence, the higher income group might have fallen through the gap. This finding contradicts previous studies where poverty was associated with non-adherence [43]. It calls for further research to understand the underlying factors.

Participants with a normal cholesterol level as comorbidity were more likely to adhere to ART than those with elevated cholesterol. A possible reason for this finding might be the pill burden, as participants with high cholesterol might experience more side effects due to the increased number of pills, thus making them less adherent or inclined to skip ART entirely. This finding is supported by another cross-sectional study in West Ethiopia, where participants with other chronic comorbidities were less adherent to ART than those without due to increased side effects and pill burden [44]. Similarly, a cross-sectional study in Ghana showed that participants with elevated cholesterol and diabetes mellitus had significantly reduced ART adherence [45].

Confidence in their self-efficacy in maintaining life was positively associated with ART adherence in this study. Self-efficacy refers to people’s confidence and beliefs in their ability to take the medication and adhere to the treatment plan [46]. In this study, self-efficacy refers explicitly to participants’ confidence in participating in healthy behaviors based on the perception that they are responsible for their own lives. This finding is consistent with other studies, demonstrating that participants with poor confidence are less likely to adhere to ART [38].

AIDS-related stigma and discrimination were negatively associated with ART adherence among stable people living with HIV in this study. Stigma, discrimination, and internal stigma were determinants of treatment adherence among cancer patients in Thailand [47]. With such experiences, people living with HIV may feel demoralized and emotionally distressed, resulting in diminished self-efficacy. Similarly, participants with poor mental health were more likely to be non-adherent than those without it. Depression has also been shown in other studies to be a determinant of ART non-adherence [31, 48]. As a result of depression, people living with HIV may feel less motivated and optimistic and perceive HIV infection as a terminal disease rather than a manageable chronic condition, thus making them unlikely to adhere to ART.

This study has several limitations. First, the study’s findings may not be generalized to all people living with HIV in Cambodia as it included only stable people living with HIV on ART. Second, we could not confirm causality due to the cross-sectional design, and reverse relationships might exist. Since this survey was part of an intervention study, future work can include exploring the changes in associated factors of ART adherence from different phases of the intervention. Such longitudinal analyses will allow us to assess the causal relationship between the factors and ART adherence over the next two years. Third, the self-reporting measures can be prone to social desirability and recall biases and possibly inflate the estimates. Forth, the lengthy questionnaire may result in a cognitive burden on the participants, leading to the use of heuristics. Lastly, we could not verify if the high level of adherence observed had corresponded to viral suppression as the viral load results were unavailable at the time of the analyses.

Despite the above limitations, we believe the study findings will provide valuable insights into the possible factors associated with ART adherence among people with HIV in Cambodia. While stable adults living with HIV are often considered to have higher ART adherence, the factors that hinder their adherence, such as low self-efficacy and high AIDS-related stigma and discrimination, could apply to those not in stable conditions to a similar extent, if not more. Further studies to explore ART adherence and its factors longitudinally during different intervention phases will address the limitation of the causal relationships between the variables in this study.

## Conclusion

This study underscores the ongoing need for comprehensive and tailored interventions that address the multifaceted challenges faced by individuals living with HIV. The factors associated with ART adherence in this study highlight the importance of public education to combat AIDS-related stigma and discrimination and community-based strategies to enhance self-efficacy and mental well-being among people living with HIV. Additionally, targeted efforts are needed to promote ART adherence among adolescents, young adults, and those with chronic comorbidities.

Cambodia has made significant strides in ensuring universal access to HIV and AIDS services, including ART. However, it remains crucial to maintain treatment adherence and provide continuous support to individuals living with HIV to achieve the ambitious third UNAIDS’ 95% target by 2030. By implementing effective strategies and collaborating with key stakeholders, we can continue to make substantial advancements in HIV treatment and care, ultimately improving the health outcomes and quality of life for those affected by the virus.

## Data Availability

The data supporting this study’s findings are available from the corresponding author (siyan@doctor.com) upon reasonable request.
